# Anti-Counterfeiting Tags Using Flexible Substrate with Gradient Micropatterning of Silver Nanowires

**DOI:** 10.3390/mi13020168

**Published:** 2022-01-22

**Authors:** Hyeli Kim, Goomin Kwon, Cheolheon Park, Jungmok You, Wook Park

**Affiliations:** 1 Department of Electronic Engineering, Kyung Hee University, Deogyeong-daero, Giheung-gu, Yongin-si 17104, Korea; khl940@khu.ac.kr; 2 Institute for Wearable Convergence Electronics, Department of Electronics and Information Convergence Engineering, Kyung Hee University, Deogyeong-daero, Giheung-gu, Yongin-si 17104, Korea; 3 Department of Plant & Environmental New Resources, Graduate School of Biotechnology, Institute of Life Science and Resources, Kyung Hee University, Deogyeong-daero, Giheung-gu, Yongin-si 17104, Korea; goomink120@khu.ac.kr; 4 Bio-MAX Institute, Seoul National University, Seoul 08826, Korea; pakchulhun@khu.ac.kr

**Keywords:** gradient pattern, maskless lithography, Ag nanowires, micropattern, UV dicing tape, anti-counterfeiting

## Abstract

Anti-counterfeiting technologies for small products are being developed. We present an anti-counterfeiting tag, a grayscale pattern of silver nanowires (AgNWs) on a flexible substrate. The anti-counterfeiting tag that is observable with a thermal imaging camera was fabricated using the characteristics of silver nanowires with high visible light transmittance and high infrared emissivity. AgNWs were patterned at microscale via a maskless lithography method using UV dicing tape with UV patterns. By attaching and detaching an AgNW coated glass slide and UV dicing tape irradiated with multiple levels of UV, we obtained AgNW patterns with four or more grayscales. Peel tests confirmed that the adhesive strength of the UV dicing tape varied according to the amount of UV irradiation, and electrical resistance and IR image intensity measurements confirmed that the pattern obtained using this tape has multi-level AgNW concentrations. When applied for anti-counterfeiting, the gradient-concentration AgNW micropattern could contain more information than a single-concentration micropattern. In addition, the gradient AgNW micropattern could be transferred to a flexible polymer substrate using a simple method and then attached to various surfaces for use as an anti-counterfeiting tag.

## 1. Introduction

Anti-counterfeiting technology is widely used in various applications in pharmaceutics, agriculture, fashion and apparel, food, winery and liquor, perfumery, cosmetics, jewelry, and electronics [1,2,3]. In order to achieve anti-counterfeiting applications, various electrical, chemical, and mechanical methods are used. With the development of IC technology in electrical approaches, small-sized electronic tags (RFID, NFC, etc.) are developed, and remote detection systems are employed. Chemically, identifying information or codes are stored as sequences of color synthesizing molecules or unique patterns formed through chemical reactions or changes in physical properties. The mechanical approach has been widely used, in which labels and tags are attached to or engraved on the products. A security pattern appears only at a specific optical wavelength or has a unique color or pattern that is difficult to reproduce [4,5,6]. In order to strengthen the identification, authentication, or tracking functions, various functional materials are used to print the texts on products or mark tags directly.

The materials widely used for these functions are luminescent materials. They have the optical property of emitting light of a different wavelength when optically excited at a specific wavelength [7,8,9]. It enables visible patterns only under UV light and is popularly used for passports and currencies. In order to create a complex anti-counterfeiting technology, methods of expressing structural color using nanoparticles or nanostructures, changing the shape of a pattern according to an angle, or changing reflectance and color using a strong magnetic field are being studied [10,11,12,13]. The environmental problems of luminescent materials and economic challenges posed by the difficulty of manufacturing nanoparticles and nanopatterns on a large scale need further improvement and attention.

Moreover, silver nanowires (AgNWs) are used in a wide range of applications, from transparent electrodes to temperature and pressure sensors, since they have advantages such as low cost, high conductivity, and flexibility [14,15,16,17,18,19]. Such applications are expanding because the manufacturing process is simple, has a relatively low cost, and patterning can be performed using various methods such as photolithography, inkjet printing, and molding. In the photolithography method, silver nanowires mixed with a photocurable polymer are patterned by UV light through the photomask. Then, silver nanowires are trapped inside the polymer using crosslinking [20,21]. The silver nanowires are dispersed in a solution and then sprayed through a nozzle of an inkjet printer to create a pattern. Pre-designed mold selectively holds and releases AgNW solution with the same pattern as the mold. In these manners, the concentration of AgNW is determined as a single value in the patterns. When different concentrations of silver nanowires can be patterned, the anti-counterfeiting tag can be more complex containing additional information.

In this study, we fabricated AgNW micropatterns in a simple way with the aid of UV dicing tape, which is used in semiconductor processing, and transferred the resulting micropattern onto various polymer substrates for anti-counterfeiting applications. Because we used a maskless lithography method, it was possible to prepare UV dicing tape with gradient adhesion characteristics through gradational UV irradiation without undergoing a complex process and to fabricate an AgNW gradient pattern by attaching and detaching it to an AgNW-coated glass slide.

The optical properties of AgNWs have received relatively less attention in previous studies. As AgNWs have low emissivity in the IR region and high light transmittance in the visible region, they can be observed only in the IR region. In addition, in the IR region, a gradational pattern can be observed depending on the concentration of the AgNWs. We exploited these optical properties of the AgNW for anti-counterfeiting.

Further, we transferred the AgNW micropattern to a flexible polymer for attaching it to various substrates for use as an anti-counterfeiting tag. It could be easily transferred to a thermosetting polymer such as Polydimethylsiloxane (PDMS) or a photocurable polymer such as Poly(ethylene glycol) diacrylate (PEGDA) [22,23], and the size of the substrate could be varied from micro- to macro-scale. We demonstrated that it could be attached to a variety of substrates such as metal, paper, and glass.

## 2. Materials and Methods

### 2.1. Materials

AgNWs (Average diameter: ~30 nm, average length: ~30 µm, dispersion solvent: Ethanol, concentration: 10 mg/mL) were purchase from 4 Science, Seongnam-si, Korea. UV dicing tape (DU 390EH) was purchased from ADTECH CO., Ltd., Gimpo-si, Korea. The PDMS elastomer for transferring the AgNWs pattern (Sylgard 184) was purchased from Dow Corning Co., Ltd., Midland, MI, USA. The poly(ethylene glycol) diacrylate 700 (Sigma-Aldrich, St. Louis, MI, USA) and 10 vol% Irgacure 1173 (BASF) as a photoinitiator was used for transferring the AgNWs pattern.

### 2.2. UV Patterning Method of UV Dicing Tape

An optical microscope (IX71, Olympus, Tokyo, Japan), UV light source (365 nm, Lightningcure LC8, Hg-Xe lamp, Hamamatsu, Shizuoka, Japan), and digital micromirror device (DMD, Texas Instruments, Dallas, TX, USA, 1024 × 768 pixels) were used for UV patterning. Various micropatterns were synthesized by projecting the patterned UV light through a 4× (NA 0.13) objective lens onto the UV dicing tape attached to a glass slide.

### 2.3. Measurement

The AgNWs pattern was imaged using an IR camera (PI640i, Optris, Berlin, Germany). A 4-point measurement method (CMT-100S, AIT Co., Ltd., Suwon-si, Korea) was used to measure the sheet resistance of AgNWs patterns.

## 3. Results and Discussion

**Figure 1:**
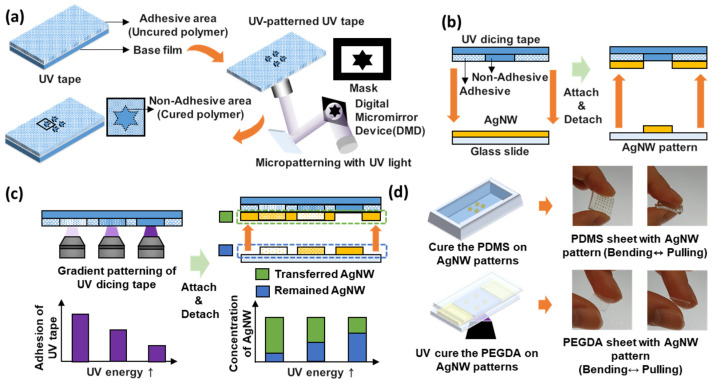
Overall process of fabricating an AgNW micropattern using UV dicing tape and transferring it to a flexible substrate. (**a**) Micropatterning of the UV tape by maskless lithography. (**b**) Micropatterning of the AgNW on a glass slide by attaching and detaching the micropatterned UV tape and AgNW-coated glass slide. (**c**) Fabrication method of the gradationally patterned AgNW on a glass slide. (**d**) Transfer of the AgNW micropattern to a flexible substrate.

Figure 1 illustrates the overall process of patterning the AgNWs on a glass slide using the UV dicing tape and then transferring it to a flexible substrate. We used a UV dicing tape, whose adhesion decreases when exposed to UV light of 365 nm wavelength at 100 mW s/cm^2^. First, the UV dicing tape was photopatterned. For this, it was temporarily attached to a glass slide, and it remained flat during the UV-patterning process. In this process, the UV light from the objective lens passes through the glass slide and irradiates the UV dicing tape, during which the photocurable adhesive applied to the UV dicing tape cures according to the pattern shape.

By using an Optofluidic maskless lithography (OFML) system [24], the UV tape attached to the glass slide was patterned to reduce the adhesiveness only in the desired area. During this process, the shape of the AgNW micropattern to be fabricated later was determined by using a digital micromirror device (DMD) mask (Figure 1a). As shown in Figure 1b, after the completion of the patterning of the UV dicing tape, the glass slide was detached from the tape.

Subsequently, the UV tape was attached to an AgNW-coated glass slide and then detached to complete the patterning in a simple manner. In the UV dicing tape, the adhesion strength of the UV-irradiated area is lower than that of the non-irradiated area, and the adhesion strength differs according to the pattern shape. Thus, the strength when the AgNWs attach to the UV-irradiated portion of the tape is weaker, and the AgNWs remain on the glass slide. The remaining AgNWs adhere to the UV dicing tape and are detached from the glass slide.

While attaching the UV-patterned UV dicing tape to the AgNW-coated glass slide, a felt squeegee was used to fix the tape uniformly without air bubbles. Thus, the tape and the glass slide spin-coated with AgNWs were attached by applying uniform pressure to the UV-patterned UV dicing tape without damage. Attaching the UV dicing tape to the AgNW-coated glass slide without air bubbles is critical for transferring patterns properly. The UV dicing tape can be attached by pressing it manually or using a load; however, if the applied strength is not uniform, the AgNWs may not detach properly, or bubbles may form.

In this study, the AgNWs were spin-coated on the glass slides. The thickness could be controlled by varying the concentration of the AgNW suspension used for spin coating or the spin-coating speed [25]. Various solvents such as ethanol, water, or isopropyl alcohol (IPA) can be used to disperse the AgNW. As the spin-coated AgNWs are not chemically bound to the glass slide, they can be easily peeled off by external adhesion. After spin coating, the solvent should be sufficiently evaporated for 1–2 h or longer. If the solvent is not removed completely, the AgNW could easily separate from the glass slide, even under a small friction force.

Figure 1c illustrates the method used for fabricating the gradient AgNW micropattern. By controlling the UV irradiation intensity of the UV dicing tape within a certain range, the curing degree of the adhesive applied to the tape could be controlled, and thus the degree of its adhesion to the AgNWs could be modulated. This control enabled the fabrication of gradational AgNW patterns.

The AgNW pattern on the glass slide can be transferred to both thermosetting and photocurable polymers. We succeeded in transferring the AgNW pattern to a polymer substrate through the following process: first, a prepolymer was loaded between the AgNW-patterned glass slide and a PDMS-coated glass slide. In this process, the thickness of the polymer substrate can be controlled using a spacer or changing the spin-coating speed used for polymer coating. After the prepolymer is loaded, the AgNW can be transferred to the polymer substrate by thermosetting or photocuring, depending on the polymer type. As the prepolymer permeates between the AgNW arrays on the glass slide and cures, the AgNW pattern is transferred to it. The AgNW pattern is located on the surface of the cured polymer. However, as the polymer permeates into the AgNW network and the system is cured together, the durability of the AgNW pattern is higher than when it is applied on a glass slide. The AgNWs could not be separated from the polymer using external force, and they did not separate even under bending or stretching (Figure 1d).

**Figure 2:**
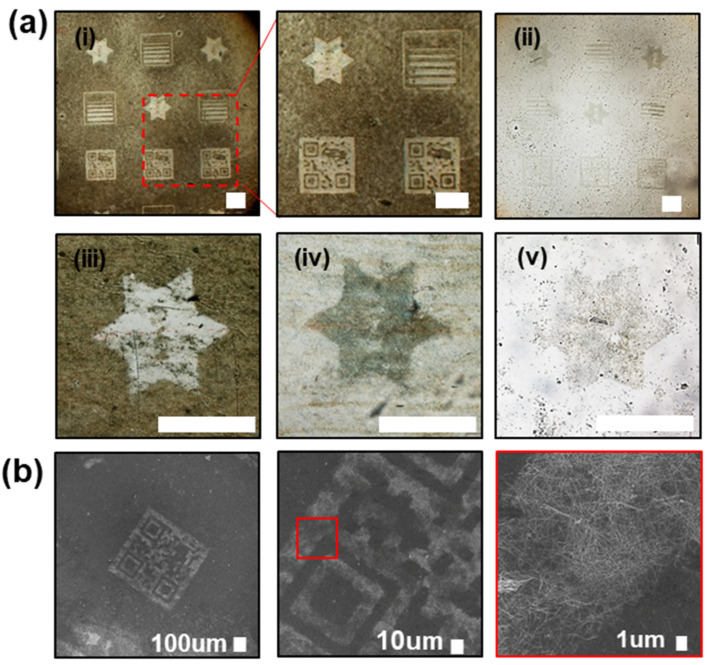
Image of an AgNW pattern fabricated using UV dicing tape. Hexagram pattern, resolution test pattern, and 2D barcode pattern were fabricated. (**a**) Bright-field image of the AgNW pattern. (Scale bar: 500 µm) (**i**) UV-patterned UV dicing tape after attachment and detachment from the AgNW-coated glass slide. AgNWs were attached to the UV dicing tape, except in the area where the adhesion was reduced by UV irradiation. (**ii**) Residual AgNW pattern on the glass slide after the attachment/detachment process. Images of the (**iii**) UV dicing tape, (**iv**) AgNW-patterned glass slide, and (**v**) AgNW pattern transferred to the PDMS substrate. (**b**) SEM image of the AgNW pattern on a glass slide, which confirms that the 2D barcode was patterned with sharp edges.

Figure 2 shows the microscopy images of the UV dicing tape and glass slide after the attaching and detaching of the UV dicing tape micropatterned using UV from the AgNW-coated glass slide, and also shows the image of the polymer after the micropattern transfer.

Figure 2a(i) shows a bright-field image of the UV dicing tape after patterning. The area observed in a relatively dark contrast contains AgNWs; it corresponds to the non-irradiated area with unaltered adhesion. On the other hand, the UV-irradiated area with reduced adhesion appears relatively bright because the amount of AgNWs attached to this area is not significant.

Figure 2a(ii) shows a bright-field image of the glass slide after the attachment/detachment process. The region that was underneath the UV-irradiated area of the tape had a relatively dark color because many AgNWs remained. Meanwhile, the region that was underneath the non-irradiated area of the UV tape appeared relatively bright because it contained fewer AgNWs.

Figure 2a(iii–v) shows the bright-field images of the UV dicing tape, glass slide, and polymer. Figure 2b shows a scanning electron microscopy (SEM) image of the AgNW micropattern on the glass slide. The enlarged image shows the well-formed QR code pattern, indicating that the micropattern was formed with high resolution. The pattern was found to have a line width of 10 μm or lower.

Although many studies were conducted on micropatterning AgNWs using various methods, such as methods that use a photomask or the inkjet printing technique, the gradient patterning of AgNWs was rarely reported. A complex fabrication process is required for fabricating heterogeneous patterns with variable AgNW concentrations using a photomask or inkjet printing. In our study, the amount of UV energy delivered to the UV dicing tape was controlled within a certain range to control the adhesion properties of the tape, and thus, AgNW patterns with varying AgNW concentrations could be fabricated in a single step.

**Figure 3:**
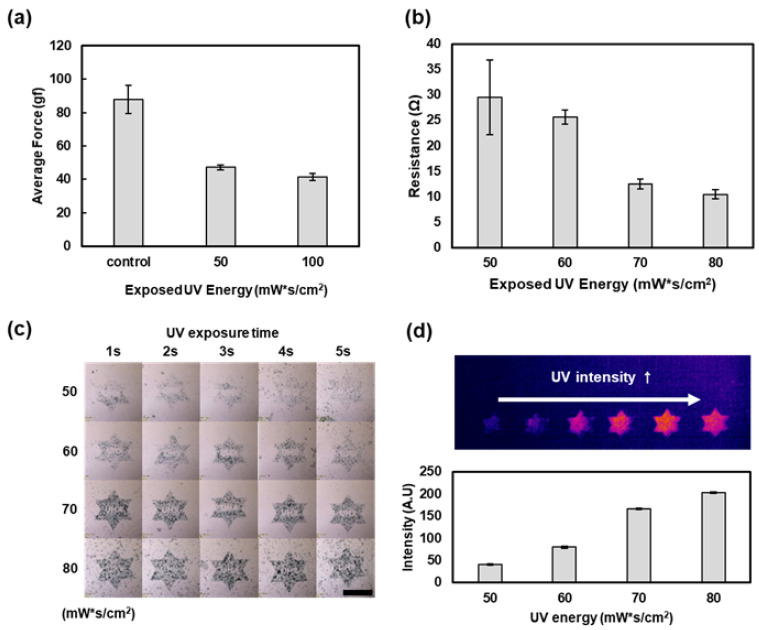
Electrical and optical properties of the AgNW pattern according to the energy of UV irradiated on the UV dicing tape. (**a**) Adhesion of the UV dicing tape according to the irradiated UV energy. (**b**) Resistance characteristics of the AgNW pattern remaining on the glass slide after attaching and detaching the UV dicing tape versus the irradiated UV energy. (**c**) Bright-field image of the AgNW pattern according to the irradiated UV energy and irradiation time. Scale bar: 1 mm (**d**) IR image of the AgNW pattern according to the irradiated UV energy and intensity.

Figure 3 shows the physical properties of the UV dicing tape according to the amount of UV light irradiated and the electrical and optical properties of the AgNW gradient pattern fabricated using the UV dicing tape. Within a certain range of the UV energy (50 to 80 mW s/cm^2^), the adhesive strength of the tape decreased in proportion to the amount of UV energy delivered to it. When the UV energy was lower than 50 mW s/cm^2^, the adhesion of the UV dicing tape did not decrease, and no patterning occurred. In addition, when the UV energy exceeded 80 mW s/cm^2^, the adhesion of the UV dicing tape was sufficiently reduced, but there was no difference in the AgNW concentration according to UV irradiation energy. Therefore, to fabricate a gradational AgNW pattern, the patterning of the UV dicing tape was performed using UV energies in the range of 50–80 mW s/cm^2^.

Further, to evaluate the change in the adhesive strength of the UV dicing tape according to UV irradiation, we performed a 90° peel test, which is a commonly used method for measuring the adhesive strength of tapes. Thus, we confirmed that the adhesive strength of the tape decreased after UV irradiation. The UV dicing tape not irradiated with UV had a peel strength of 87.8 gf, while the UV-irradiated tape had peel strengths of 47.3 and 41.3 gf when irradiated with 50 and 100 mW s/cm^2^ of UV, respectively. The 90° peel test is used to measure the force applied for separating the UV dicing tape at 90° after attaching it to a glass slide, and this force is expected to correlate with the force applied to separate the UV tape from the AgNW-coated glass slide (Figure 3a).

Furthermore, the UV energy was controlled within the range of 50–80 mW s/cm^2^ to fabricate a 3 cm × 3 cm square pattern. The electrical resistance of the AgNWs remaining on the glass slide was measured using a four-probe measurement method. The electrical resistances of the AgNW patterns fabricated using 50, 60, 70, and 80 mW s/cm^2^ UV energy were 29.4, 25.6, 12.4, and 10.4 ohm, respectively. In summary, when the UV dicing tape was irradiated with a large amount of UV, the amount of residual AgNWs on the glass slide increased, and the electrical resistance was low (Figure 3b).

Next, the UV tape was patterned using a DMD mask with the same pattern, and the total irradiation time was varied between 1 and 5 s, while the UV energy was varied between 50 and 80 mW s/cm^2^ (Figure 3c). It was confirmed that the AgNW patterns contained similar AgNW contents when the total UV energy was the same, although the UV irradiation time was different. Figure 3d shows the image of the gradational AgNW pattern obtained using an IR camera, which reveals that the measured image intensity varied according to the AgNW concentration in the pattern.

**Figure 4:**
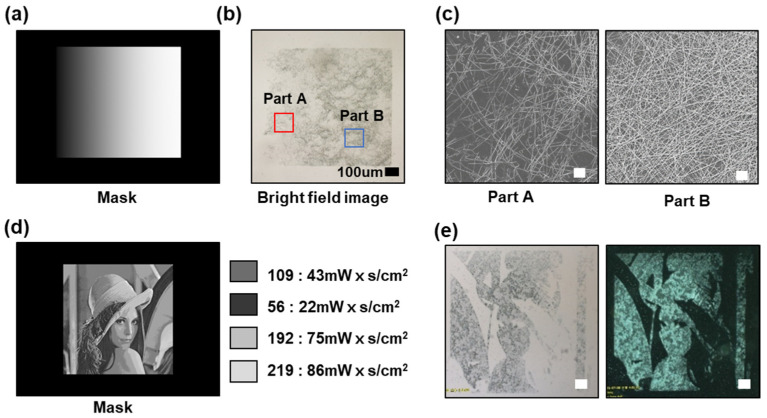
Gradient AgNW micropattern fabricated using a gradationally patterned DMD photomask. (**a**) DMD photomask used for gradient AgNW micropatterning. (**b**) Bright-field image of an AgNW micropattern fabricated using the mask shown in (**a**). (**c**) S SEM images of the areas with a relatively low concentration of AgNWs (Part A) and relatively high concentration of AgNWs (Part B) in the AgNW pattern of (**b**) (Scale bar: 1 µm). (**d**) Photomask used for micropatterning a Lena image and the grayscale value of the image. (**e**) Bright-field and dark-field images of the AgNW-micropatterned Lena image (Scale bar: 200 µm).

We previously demonstrated that the adhesive force of the UV dicing tape could be controlled within a certain range depending on the amount of UV energy delivered to the UV dicing tape, and it was confirmed that the concentration of the residual AgNWs on the glass slide after the attachment/detachment process could also be controlled. By using these properties of the UV dicing tape, gradation was implemented within a single pattern, as shown in Figure 4, and AgNW gradational micropatterns were fabricated using DMD photomasks with a simple square gradation pattern and a relatively complex Lena image pattern.

Figure 4b shows a bright-field image of the AgNW micropattern fabricated using the mask shown in Figure 4a. SEM images of different regions of this pattern with different AgNW concentrations are shown in Figure 4c, which demonstrates the difference in the density of the AgNWs in different regions. Figure 4d shows a DMD mask with a Lena image, which is a complex gradient pattern. The amount of UV energy irradiated varies according to the grayscale of the mask. Both bright-field and dark-field images of the AgNW micropattern fabricated using this DMD mask are shown in Figure 4e for clear visualization of the pattern.

Thus, we succeeded in fabricating gradational AgNW micropatterns without a complex process. Both simple and complex patterns could be generated, and the processing time of this process is the same as that required for a single-concentration pattern.

**Figure 5:**
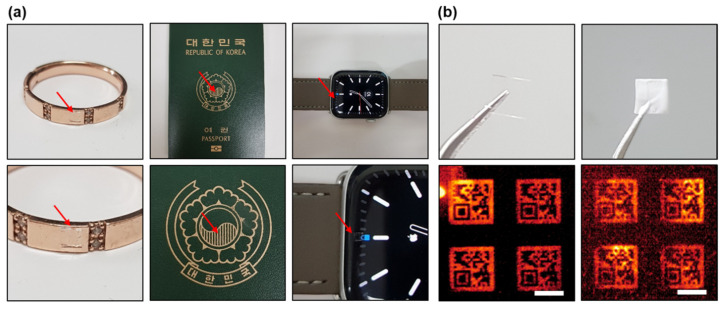
An example of the use of AgNW micropatterns in anti-counterfeiting. The flexibility of the polymer substrate and the optical properties of AgNWs were used. (**a**) Images show an AgNW micropattern transferred to a polymer substrate and then placed on various surfaces. Because of its high transparency in the visible light region, the micropattern could be attached to various surfaces for anti-counterfeiting. It was attached to a ring, passport, and display panel of a watch. (**b**) Images of an AgNW-micropatterned polymer substrate before and after coating with paint. The code could be clearly observed in the IR-irradiated area even when it was completely covered with the paint (Scale bar: 1 mm).

Figure 5 illustrates a method of exploiting the flexibility of the polymer substrate and the optical properties of AgNWs for anti-counterfeiting. We transferred the AgNW micropatterns onto a transparent flexible polymer substrate that can be attached to a variety of surfaces to be used as an anti-counterfeiting tag.

AgNWs have high transparency in the visible region owing to their high light transmittance and low emissivity but can be clearly observed under IR light. By using these properties, a hidden code for anti-counterfeiting or an AgNW-patterned polymer film for a wearable device was fabricated and imaged in the visible and IR regions.

In our study, AgNW micropatterns could be fabricated in a simple way, and various patterns, such as QR codes and Lena images could be fabricated with gradient AgNW concentrations. The obtained micropattern could also be transferred to various types of polymer substrates of different sizes. The substrates could have flexibility or stretchability depending on the type of polymer. Moreover, AgNW-micropatterned polymer substrates can be fabricated in various forms, such as microparticles or films.

In order to demonstrate the use of the optical properties of AgNWs in anti-counterfeiting, we transferred the AgNW micropattern in the shape of a micro QR code to a PDMS substrate as an example. As shown in Figure 5a, the AgNW micropattern was transferred to a PDMS substrate and then attached to a ring, passport, and smartwatch. Figure 5b shows images of an AgNW-micropatterned PDMS substrate before and after being painted with aerosol paint. As AgNWs have high light transmittance in the visible region, the pattern is relatively transparent but can be observed on a dark background. Therefore, to completely hide the pattern from being observed under visible light, the polymer substrate can be painted. Although the brightness of the IR image may decrease depending on the thickness of the paint coating, the pattern could be identified, as shown in Figure 5b.

## 4. Conclusions

In this study, we developed a simple method for the gradient micropatterning of AgNWs using UV dicing tape and the subsequent transfer of the micropattern to a polymer substrate. This method does not depend on the type of substrate or the specification of the AgNWs and provides a gradient pattern without a complex process. UV dicing tape has an advantageous property of decreasing adhesive strength with UV irradiation, and it has been widely used in semiconductor processes but rarely applied in micropatterning. Moreover, compared to the patterning method using a liquid adhesive prepolymer, our method of using UV dicing tape has the advantage of being simple and less toxic. In addition, it was possible to prevent phenomena such as blurring or air bubbles that may occur during UV patterning with the use of a liquid prepolymer. Moreover, this method is scalable, and the pattern can be transferred to various types of polymers. Another advantage of using UV dicing tape is that its adhesion can be controlled by changing the UV energy and intensity. By using this method, we were able to fabricate gradient AgNW micropatterns without complex processes. AgNW micropatterning is a technology that is actively studied in various fields, such as in the development of sensors and flexible devices, in addition to being studied for anti-counterfeiting because of the various advantages of the AgNWs, such as low price, high flexibility, high light transmittance, and high electrical conductance. The AgNW micropatterning technique developed in this study can be utilized in various fields.

In addition, the AgNW gradient micropattern produced in our study is expected to be applied in the production of AgNW micropatterns with different electrical conductivities in addition to anti-counterfeiting tags. We patterned the UV dicing tape over an area of several centimeters (lab scale), but it will be possible to manufacture it over a large area through the roll-to-roll process in the future. In addition, the tape can be applied for micropatterning various materials such as poly (3,4-ethylenedioxythiophene) PEDOT and carbon nanofibers, in addition to AgNWs.
